# Retention of teeth in the fracture gaps of the mandible: a retrospective analysis

**DOI:** 10.1007/s00784-023-05218-5

**Published:** 2023-08-23

**Authors:** Linus Josef Walker, Sabine Koba, Aktug Demiroglu, Nikola Saulacic, John-Patrik Burkhard

**Affiliations:** 1 Department of Cranio-Maxillofacial Surgery, Inselspital, Bern University Hospital, University of Bern, CH-3010 Bern, Switzerland; 2 Limmat Cleft and Craniofacial Centre, CH-8005 Zurich, Switzerland

**Keywords:** Mandibular fractures, Tooth, Fracture line

## Abstract

**Objectives:**

Since the introduction of miniplate osteosynthesis and the use of prophylactic antibiotics, the complication rate related to the teeth in the fracture gap has significantly decreased. Currently, there are still no established guidelines for the management of such teeth in mandibular fracture lines. However, the long-term viability of these teeth within the fracture gap remains uncertain. Therefore, this study aimed to assess the survival rate of teeth located within the mandibular fracture line and evaluate related follow-up treatments over a minimum period of one year.

**Materials and methods:**

This retrospective study examined 184 patients who underwent surgical treatment for mandibular fractures between January 2018 and December 2021. A total of 189 teeth located in the fracture line were analyzed. Clinical and radiological parameters were collected, including patient age and gender, fracture etiology and location, intraoperative tooth treatment, as well as complications related to both the fracture and the affected teeth in long term.

**Results:**

Most of the examined teeth remained uneventful, with postoperative tooth-related complications seen in 14 (7.4%) teeth. The most common complications were symptomatic apical periodontitis (*n* = 9, 4.8%) and increased tooth mobility (*n* = 3, 1.5%). A correlation was found between complications and trauma-related tooth luxation (p = 0.002, OR = 15.2), as well as prior teeth connected to retainers or orthodontic appliances (p = 0.001, OR = 10.32).

**Conclusion:**

Tooth-related complications are rare when intact teeth are retained within the fracture gap. Therefore, unless there is a definitive intraoperative indication for extraction, it is recommended to preserve the teeth in the fracture line.

**Clinical relevance:**

Intact teeth in the fracture line of the mandible should not be primarily extracted.

## Introduction

Epidemiological data regarding mandibular fractures exhibit variation across different countries. In Switzerland, these fractures are most frequently observed in the younger population, specifically among individuals aged 16 to 29 years [1]. Men have a notably higher incidence of mandibular fractures, often attributable to traffic or sports accidents. The condylar region and the symphyseal/parasymphyseal area are the most commonly affected sites, which remains consistent even among patients aged 65 years and above [1, 2]. Although the high involvement of tooth-bearing areas (56 to 69%) primarily affects younger patients, an adequate treatment decision is necessary to preserve the teeth, avoid subsequent treatments and follow-up costs [3].

The presence of a tooth within a fracture line always implies a connection between the oral cavity and the mandibular bone, courtesy of the periodontal ligament [4]. Such a tooth can suffer various forms of damage at the fracture site, including exposure of the root surface, (sub)luxation, root fracture, or even complete avulsion, all of which can potentially result in tooth devitalization. The presence of preexisting pulpal, periodontal, or periapical pathologies can further exacerbate these issues [4]. All of these problems, either individually or in combination, can lead to infections and/or impaired healing [4, 5]. Moreover, impacted teeth in particular might impede the proper repositioning of the fracture, resulting in inadequate immobilization or misalignment, leading to malocclusion. The extraction of such teeth carries the risk of causing additional trauma to the surrounding bone tissue, particularly when the fracture fragments are highly mobile. This poses a significant challenge from a technical perspective [6]. Consequently, the decision of whether to remove or retain teeth located in the fracture line remains a topic of ongoing discussion [3, 4, 7].

Prior to the advent of antibiotics and (semi-)rigid fixation methods, authors agreed that all teeth within a fracture line should be extracted to eliminate potential sources of infection, regardless whether they were vital or not [8]. This approach was attributed to the limitations of intermaxillary fixation and its inadequate stability. However, with the introduction of stable three-dimensional fixation using miniplate osteosynthesis, which requires precise anatomical reduction, the routine removal of teeth has become significantly less necessary [9–12]. Despite extensive research in the field of facial traumatology, the existing literature still lacks clear guidance regarding the management and long-term outcomes of teeth located within the fracture gap [3, 10–12]. The question of whether to extract or retain such teeth in cases of mandibular fractures remains a subject of controversy. Therefore, the objective of this study was to investigate epidemiological data, complications, and long-term survival rates of teeth preserved within the mandibular fracture line.

## Materials and methods

A total of 184 patients with 189 teeth in the fracture line who were admitted to the Department of Oral and Maxillofacial Surgery at the University Hospital of Bern, Switzerland, between January 2018 and September 2021, were included in this study. The selection focused on patients with fractures in the dentate region of the mandible, and their health-related data and radiographs were reviewed. This retrospective study was conducted in compliance with the Declaration of Helsinki and approved by the Cantonal Ethics Committee of Bern (Nr. 2021–00013). The study also adheres to the STROBE guidelines (Strengthening the Reporting of Observational Studies in Epidemiology).

The evaluation involved assessing the selection of teeth based on the location and type of mandibular fracture. For each case, the number and location of the affected teeth were analyzed, along with their condition (e.g., restored, endodontically pretreated, fractured as a result of the trauma, periodontal damage). Additionally, the teeth were classified based on their eruption state, such as retained, impacted, or in occlusion.

**Fig. 1:**
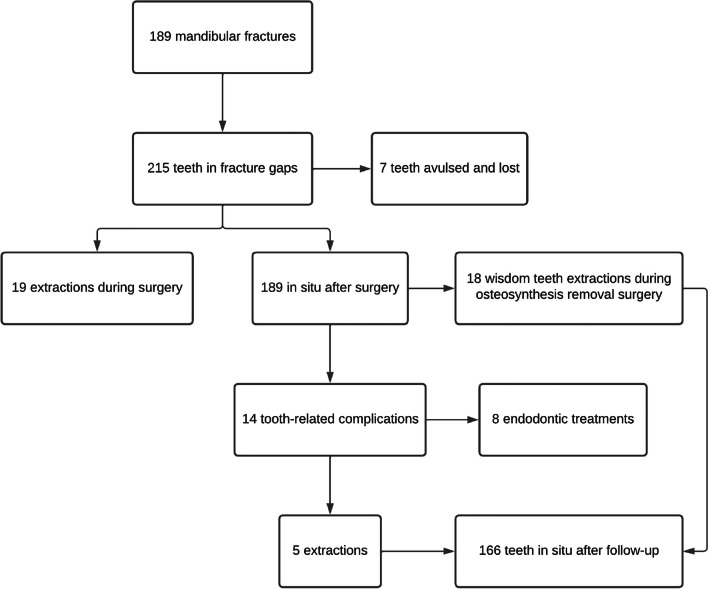
Flowchart depicting the progression of teeth within the fracture gap

Relevant information, including age, gender, and type of injury, was collected. The mechanism of trauma was further categorized into falls, traffic accidents, sports-related injuries, assaults, and others. Fracture-specific parameters were obtained by examining orthopantomographs (OPGs) and computed tomography (CT) scans. The traumas were classified based on whether only the mandible was affected or if additional facial cranial bones were involved, as well as the severity of the fractures, either simple or comminuted. The number of fracture lines in the mandible was determined on the basis of dental landmarks (Fig. 1). Additionally, the duration between the accident and fracture treatment, as well as the duration of antibiotic therapy were recorded.

Fracture treatment followed the Association of the Study of Internal Fixation (AO) guidelines and involved intermaxillary fixation (IMF) using a modified Schuchardt splint or IMF screws, as well as osteosynthesis through an intraoral approach using two miniplates or a 3D plate. In accordance with our internal guidelines, the primary objective is to retain a tooth located within the fracture gap, except when it is impacted by periodontal damage that poses an increased risk of infection. Moreover, teeth exhibiting irreparable defects, such as non-restorable caries-related issues or deep subgingival/vertical crown-root fractures caused by the trauma itself, are primarily extracted. Teeth that interfered with adequate reduction of the fracture were extracted, unless their removal would impede the fracture healing process.

The postoperative procedures adhered to the hospital's internal guidelines, with the continuation of antibiotic therapy typically involving three intravenous doses over 24 h postoperatively. Patients were instructed to maintain good oral hygiene, and additional oral cleansing support was provided through the administration of chlorhexidine digluconate three times daily.

Complications following fracture repair were classified into the following categories: infection (with or without the need for reoperation), wound dehiscence, hypesthesia, malocclusion, mandibular deviation, fracture of the osteosynthesis material, and nonunion. All surgical reinterventions were defined as major complications. Follow-up examinations were conducted at specific intervals, including 1 week, 3 weeks, and 6 weeks postoperatively until the removal of splints or IMF screws. Subsequently, follow-up visits were scheduled at 3- to 6-month intervals, totaling up to 12 months. During the one-year follow-up period, any tooth-specific complication including changes in tooth vitality, additional root canal treatment, or need for extraction (with potential replacement by an implant) were documented.

The statistical analysis was performed using IBM® SPSS® Statistics (Version 28.0.0.0 for macOS). Mean and standard deviation values were calculated for continuous variables. Chi-squared tests were employed to compare categorical independent variables with the primary binary outcome (tooth-related complications). Logistic regressions were utilized to compare non-binary categorical independent variables with the primary outcome. A significance level of *p* < 0.05 was established, and all estimations employed 95% confidence intervals.

## Results

Out of a total of 184 patients who underwent surgical treatment for mandibular corpus or angle fractures, a combined total of 208 teeth located in the fracture line met the inclusion criteria for this study (Fig. 1). Among the patients, 138 were male (75%) and 46 were female (25%). The age of the patients ranged from 17.7 to 94.6 years, with a mean age of 37 years [standard deviation (SD) ± 19.2]. The most common causes of mandibular fractures were falls (37.5%, *n* = 69), followed by assault (29.3%, *n* = 54), bicycle accidents (19.6%, *n* = 36), sports-related injuries (11.4%, *n* = 21), and traffic accidents (2.2%, *n* = 4). Detailed compilation in Table 1.

**Table 1:** Baseline characteristics

	Reference
	All
	*n (%)*
Age at injury, mean [SD], y	37 [± 19]
Sex	
Female	46 (25%)
Male	138 (75%)
Fracture mechanism	
Fall	69 (37.5%)
Assault	54 (29.3%)
Bicycle accidents	36 (19.6%)
Sports	21 (11.4%)
Traffic accident	4 (2.2%)
Isolated trauma of the mandible	143 (77.7%)
Time from diagnosis to surgery, mean [SD], d	2 [± 1.8]
Time of antibiotic prophylaxis, mean [SD], d	4 [± 3.9]
Types of tooth-related complications	
Apical periodontitis	9 (64.2%)
Increased mobility	4 (28.6%)
Hypersensitivity	1 (7.1%)
Postoperative tooth intervention needed	
Endodontic treatment	8 (4.2%)
Extraction (prophylactic extractions included)	23 (12.2%) *

* During the removal of osteosynthesis material, a total of 18 teeth were extracted as a preventive measure

Operative fracture treatment was performed within an average of 2 days (SD ± 1.8) after the radiological diagnosis of the fracture and patient hospitalization. In the majority of cases (77.7%, *n* = 143), mandibular fractures were isolated, while in 22.3% of cases (*n* = 41), they were associated with fractures of the facial skeleton. Postoperative antibiotic treatment was administered for an average of 4 days (SD ± 3.9).

**Fig. 2:**
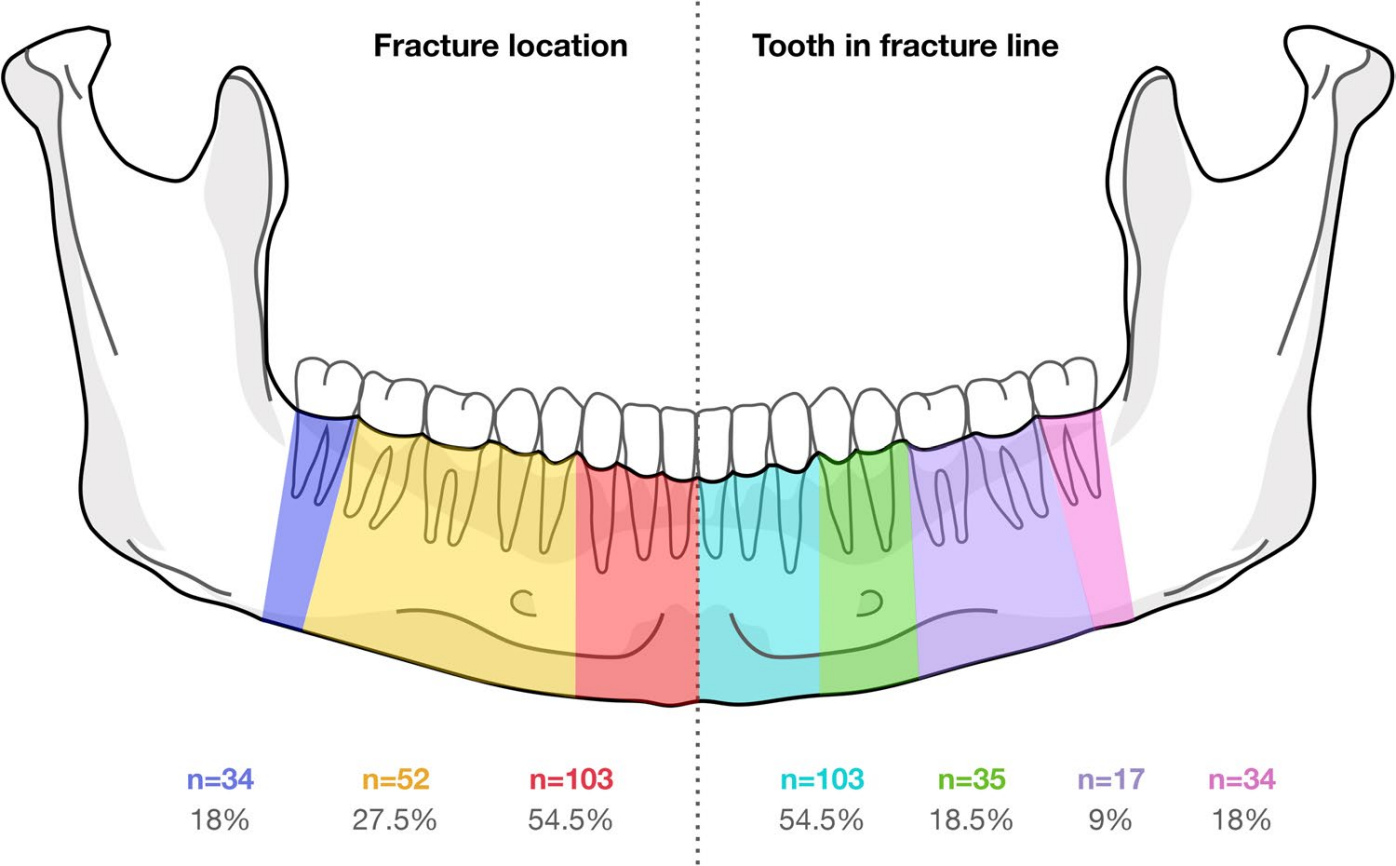
Visualization of the location of the fractures and the affected teeth within the fracture gap

Out of the 189 teeth in the fracture line following the mandibular fracture treatment, the distribution was as follows: 103 incisors and canines (54.5%), 35 premolars (18.5%), 17 molars (9%), and 34 wisdom teeth (18%), as shown in Fig. 2. The majority of these teeth (87.8%, *n* = 166) were erupted and aligned in the occlusion plane. Among them, 22 teeth (11.6%) were retained, and one tooth (0.6%) was impacted. There were six teeth (3.2%) that were luxated, and five teeth (2.6%) had either coronal or long axis root fractures. Prior to the accident, only 12 teeth (6.3%) had fillings, and two cases (1.1%) already had endodontic fillings.

Among the initial 208 teeth observed within the fracture gap, 19 teeth (9.1%) required extraction during the procedure due to reasons such as irreparable tooth fractures (*n* = 12), interference with proper fracture reduction (*n* = 3), pre-existing periodontal damage, or caries (*n* = 4). Among the extracted teeth, five (2.4%) were wisdom teeth. Additionally, seven teeth (3.3%) were chipped and lost as a result of the trauma.

### Fracture-related complications

Out of the 184 patients analyzed, a total of 83 fracture-related complications were observed, with some patients experiencing multiple complications, accounting for 35.3% (*n* = 65) of the patients. Among these complications, the most prevalent was hypoesthesia of the inferior alveolar nerve (IAN), occurring in 42.2% of cases (*n* = 35). Malocclusion was reported in 26.5% of all patients (*n* = 22). However, it is noteworthy that these instances showed improvement or complete recovery over time. Other complications included wound dehiscence (9.6%, *n* = 8), infection requiring abscess relief (3.6%, *n* = 3), infection treated with antibiotics (4.8%, *n* = 4), non-union requiring reoperation (6%, *n* = 5), mandibular deviation (4.8%, *n* = 4), fracture of osteosynthesis material requiring reoperation (1.2%, *n* = 1), and orofacial pain caused by tendomyopathy (1.2%, *n* = 1).

Among the nine cases (4.8%) of major complications, three required abscess opening due to surgical site infections. In two cases (1%), the complications were attributed to the teeth that were left in place, leading to subsequent extraction or endodontic treatment. Among the five cases of non-union, two teeth in the fracture gap were prophylactically extracted during surgery due to evident periodontal damage and the potential for infection. Additionally, one case required reoperation due to inadequate fracture reduction. In the remaining two cases, the lack of root treatment in a non-vital tooth may have contributed to the nonhealing of the fracture in one case (0.5%), especially considering that the patient had the tooth extracted at a later stage. However, the precise cause of non-healing remains unclear in the latter case.

### Tooth-related complications

Tooth-related complications were observed in 13 patients (7.1%) involving a total of 14 teeth (7.2%). The most common complications were symptomatic apical periodontitis (64.2%, *n* = 9), increased mobility (28.6%, *n* = 4), and hypersensitivity (7.1%, *n* = 1). Based on the initial count of 189 teeth in the fracture gap, it was found that 8 teeth (4.2%) required endodontic treatment, and 5 teeth (2.6%) needed to be extracted.

During the follow-up period, a total of 23 teeth (12.2%) originally located in the fracture gap had to be extracted. Among them, 18 asymptomatic wisdom teeth (9.5%) were extracted as a preventive measure during the removal of osteosynthesis material [13], while 5 teeth were deemed not viable for preservation due to crown fractures (*n* = 3) or symptomatic apical periodontitis (*n* = 2). Additionally, 8 symptomatic or non-vital teeth (4.2%; 3 incisors, 4 canines, and 1 premolar) underwent or were scheduled for endodontic treatment. Interestingly, all dental interventions performed postoperatively were required for mandibular fractures that were initially dislocated. The logistic regression analysis revealed a significant correlation between the occurrence of tooth-related complications and tooth luxation at the time of trauma (p = 0.002, OR 15.2), as well as the presence of orthodontic appliances or retainers (p = 0.001, OR 10.3). However, factors such as sex, fracture type, fracture (dis)location, time from diagnosis to surgery, duration of postoperative antibiotic treatment, type of tooth in the fracture gap, crown or root fractures, occlusion status, and pretreatment with fillings or endodontic treatment did not show statistically significant correlations with postoperative tooth-related complications throughout the one-year follow-up period. Detailed compilation in Table 2.

**Table 2:** Baseline, intraoperative and postoperative variables with tooth-related complications

	Reference	tooth-related complications				
	*All*	*No*	*Yes*	*p*	*OR*	*Confidence interval*
	*n (%)*	*n (%)*	*n (%)*			
Sex						
Female	55 (29.1%)	50 (90.9%)	5 (9.1%)	0.571	1.389	[0.444—4.349]
Male	134 (70.9%)	125 (93.3%)	9 (6.7%)			
Type of fracture						
not comminuted	158 (83.6%)	146 (92.4%)	12 (7.6%)			
comminuted	31 (16.4%)	29 (93.5%)	2 (6.5%)	0.824	0.839	[0.178—3.95]
not dislocated	77 (40.7%)	73 (94.8%)	4 (5.2%)			
dislocated	112 (59.3%)	102 (91.1%)	10 (8.9%)	0.336	1.789	[0.54—5.928]
Fracture location						
median/paramedian	103 (54.5%)	92 (89.3%)	11 (10.7%)			
body	52 (27.5%)	49 (94.2%)	3 (5.8%)	0.321	0.512	[0.136 – 1.922]
angle	34 (18%)	34 (100%)	0 (0%)			
Time from diagnosis to surgery						
< 3 days	156 (82.5%)	145 (92.9%)	11 (7.1%)			
> 3 days	33 (17.5%)	30 (90.9%)	3 (9.1%)	0.684	1.318	[0.347—5.013]
Postoperative antibiotic duration						
< 3 days	92 (48.7%)	86 (93.5%)	6 (6.5%)			
> 3 days	97 (51.3%)	89 (91.8%)	8 (8.2%)	0.651	1.288	[0.429—3.867]
Type of tooth affected						
front	103 (54.5%)	92 (89.3%)	11 (10.7%)			
premolar	35 (18.5%)	33 (94.3%)	2 (5.7%)	0.393	0.507	[0.107—2.408]
molar	17 (9%)	16 (94.1%)	1 (5.9%)	0.548	0.523	[0.063—4.332]
wisdom tooth	34 (18%)	34 (100%)	0 (0%)			
Fracture related tooth damage						
none	178 (94.2%)	167 (93.8%)	11 (6.2%)			
crown/root fracture	5 (2.6%)	5 (100%)	0 (0%)			
luxation	6 (3.2%)	3 (50%)	3 (50%)	0.002	15.182	[2.739—84.163]
Tooth in fracture line related factors						
in occlusion	166 (87.8%)	152 (91.6%)	14 (8.4%)			
retained	22 (11.6%)	22 (100%)	0 (0%)			
impacted	1 (0.6%)	1 (100%)	0 (0%)			
no pretreatment	165 (87.3%)	155 (93.9%)	10 (6.1%)			
retainer/orthodontic appliance	10 (5.3%)	6 (60%)	4 (40%)	0.001	10.333	[2.504—42.647]
pretreated with filling	12 (6.3%)	12 (100%)	0 (0%)			
pretreated with endodontic	2 (1.1%)	2 (100%)	0 (0%)			

## Discussion

This study aimed to assess the survival rate of teeth located in the mandibular fracture gap following regular fracture reduction, to track the outcomes of preserved teeth and analyze their correlation with fracture-related and tooth-related factors that affect the prognosis of teeth in mandibular fracture lines.

Researchers hold varying opinions on the optimal treatment approach for teeth located in mandibular fracture lines. The debate centers around whether primary extraction should be pursued during fracture reduction to minimize postoperative fracture-related complications [3, 9, 12–16]. With the introduction of (semi)rigid fixation techniques involving osteosynthesis plates, along with the administration of prophylactic antibiotics, has been instrumental in significantly reducing the frequency of such complications in fracture management [9, 17]. In our study, the occurrence of postoperative fracture-related complications was found to be as frequent in the presence of a devitalized tooth in the fracture gap as in the case of prophylactic extraction of the tooth because it was periodontally pre-damaged. Both situations were represented with a frequency of up to 1%. This finding is supported by another study, which found no statistical evidence to suggest that removing teeth in fracture lines reduces morbidity when compared to cases where teeth were retained [18].

Due to the close proximity of teeth in the fracture site to the jawbone through the periodontal gap, mobile teeth are often considered potential sources of infection [19]. Dislocated fractures are also more frequently associated with dental problems, as the vascular nerve bundle is often disrupted [4]. Therefore, some authors suggest the prophylactic removal of these teeth [16, 20]. In our study, a total of nineteen teeth were extracted as a preventive measure, primarily due to irreparable damage, such as deep crown and root fractures caused by the initial trauma. Preserved teeth that exhibited tooth-related complications were observed in cases where teeth were dislocated due to trauma in the fracture gap. This can be attributed to the likely disruption of the vascular nerve bundle. However, these findings do not support the notion of prophylactic extraction, particularly considering that adequate retention and prompt endodontic treatment can ensure the preservation of teeth in the dental arch. Our study demonstrated that such complications occurred in less than 1% of cases. Sufficient stabilization of mobile teeth can be achieved by integration in the Schuchardt splint, as demonstrated in our cases. These must be closely followed up postoperatively and, if necessary, treated endodontically in the event of devitalization. This applies particularly to initially luxated teeth and teeth in fractures with dislocation.

A consensus on extraction of teeth in mandibular fracture lines has been achieved for fractured roots, teeth which impede fracture reduction, exhibit extensive periapical radiolucencies, or are distracted by fractures with extensive periodontal damage resulting in deep pockets [3]. Significant differences in the occurrence of dental problems were observed in teeth with a bony displacement exceeding 3 mm. The location of the fracture line in relation to the tooth apex seems not to affect the occurrence of dental problems [21].

Recent study [17] have indicated that preserving teeth in the fracture lines may lead to lower complication rates which is consistent with the results of the present study. We recommend retention of teeth worthy of preservation in the fracture gap with close postoperative monitoring and, if necessary, early endodontic treatment. Although this study has some limitations, such as the small number of cases and retrospective nature, it provides valuable insight into this topic. To the best of our knowledge, no study has observed teeth in the fracture gap over a long follow-up time. It is worth noting that over a one-year follow-up period, extremely few tooth-related complications occurred, which mainly ended in extraction or endodontic treatment with virtually no effect on fracture healing. However, future studies should aim to address these limitations by increasing the sample size and conducting a prospective analysis. Conducting such studies will contribute to a more comprehensive understanding of the issue.

## Conclusion

Tooth-related complications in the mandibular fracture line seem to be extremely rare when the teeth are undamaged, properly preserved, and closely monitored. As a result, the extraction of teeth in the mandibular fracture line may only be considered in cases where preservation is not feasible due to significant tooth damage.
